# Independent measurement of face perception, face matching, and face memory reveals impairments in face perception and memory, but not matching, in autism

**DOI:** 10.3758/s13423-023-02304-3

**Published:** 2023-06-13

**Authors:** Mirta Stantić, Katie Brown, Eri Ichijo, Zoë Pounder, Caroline Catmur, Geoffrey Bird

**Affiliations:** 1 Department of Experimental Psychology, University of Oxford, Oxford, UK; 2 Department of Psychology, King’s College London, London, UK; 3 School of Psychology, University of Birmingham, Birmingham, UK

**Keywords:** Face matching, Face perception, Face memory, Face recognition, Autism

## Abstract

Multiple psychological processes are required in order for a face to be recognised from memory. However, when testing face memory using tasks such as the Cambridge Face Memory Task (CFMT), it is rare for studies to attempt to account for individual differences in face perception and face matching in order to isolate variance in face memory specifically. In Study 1, the Oxford Face Matching Test (OFMT) was used to assess face matching and face perception in a large sample of participants (N = 1,112). Results revealed independent contributions of face perception and matching to CFMT performance, and these results replicated with the Glasgow Face Matching Test. In Study 2, the same procedure was used to test face perception, face matching and face memory in a group of 57 autistic adults and a matched neurotypical control group. Results revealed impaired face perception and memory in the individuals with autism, but intact face matching. Face perception may therefore act as a potential intervention target for individuals with autism who exhibit face recognition impairments.

## Introduction

Psychological models of the processes by which we recognize our conspecifics from their faces acknowledge that face recognition can be broken down into a number of sub-processes (Bruce & Young, 1986; Haxby et al., 2000). For example, in the classic model by Bruce and Young (1986), recall of an individual’s name upon viewing a photograph of their face requires multiple steps. Initially, a perceptual representation of a face is formed that consists of both view-dependent and more abstract perceptual representations of the face. These representations activate face recognition units – representations of known faces – according to the degree of similarity between the face in the photograph and the stored face representations. In turn, face recognition units activate person identity nodes that contain identity-specific semantic codes (information such as the person’s occupation, where they live etc.), which go on to activate the appropriate name code for the presented face. Bruce and Young (1986) also highlighted the importance of decision processes in their model, especially the ability to determine whether an image of a face matches a particular facial identity. This process is needed when deciding whether two photographs of a face depict the same person, and when deciding whether the face of a person matches any of the facial identities one has previously learned.

Turning from theory to empirical methods, measures of face processing can be split into three types based on the specific aspects of face processing that they aim to measure. The first category contains tasks assessing face detection or face attention – the ability (or propensity) to identify, and to attend to, faces among non-face distractors. The second category contains tasks assessing face perception, which are generally described as assessing the degree to which a veridical three-dimensional (3-D) perceptual representation of a face can be derived from sometimes limited information (e.g., a two-dimensional (2-D) image from one viewpoint only). In practice, such tasks are commonly (but not exclusively) tests of face matching, in which two facial images are presented concurrently or in quick succession, and participants are required to decide whether the facial images are of the same individual, or from different individuals. The third category contains face recognition tests, with individuals required to recognize famous faces, or faces they have previously been exposed to. Note that this category of test may be further sub-divided into those that require a simple judgement of whether a face has been seen before (“old/new” judgement), and those that require recall of a label (such as a name or other semantic information) associated with a face. Although terms are not used consistently, the former are more likely to be called *tests of face recognition* and the latter *tests of face memory*. It is clear, however, that both require faces to be stored in memory.

With respect to the third category, although face recognition tests (e.g., the Cambridge Face Memory Task (CFMT); Duchaine & Nakayama, 2006) are of use when identifying individuals who exhibit severe difficulties with face recognition (i.e., individuals with prosopagnosia), they are not ‘process pure’. When an individual performs poorly on these tests compared to individuals with typical face recognition ability, it is difficult to truly determine the precise psychological process (or combination of processes) that may contribute to this poor performance. For example, if an individual fails to recognize famous faces, or faces they have previously seen, they may have difficulties with building a perceptual representation of the face, with determining whether a stimulus face matches a face stored in memory, or with face memory (storage and retrieval of face data) specifically. Therefore, poor performance on face memory tasks, such as the CFMT, may be driven by impairments in one of face perception, face matching or face memory, or in a combination of these processes.

In a similar fashion, face matching tasks (e.g., Glasgow Face Matching Test (GFMT); Burton et al., 2010) are also not process pure. In these tasks, a participant may fail to recognize two facial images are of the same individual because they cannot form an accurate perceptual representation of the faces (i.e., they have difficulties with face perception), or because, even though they can form accurate perceptual representations, they are poor at *deciding* whether the two faces are of the same individual or of different individuals (i.e., they have difficulties with the psychological process of face matching). Thus, individuals may perform poorly on face matching tasks because they have impairments in face perception, face matching, or both.

The psychological process of face matching we characterise by the use of a model of how faces may vary within and between individuals (e.g., an individual’s face changes colour slightly as they tan, but their face skeletal structure is unlikely to change) to make the decision as to whether the extent and nature of similarity between two faces is such that they must conclude they belong to the same or different identities. For clarity, we distinguish between the *psychological process* of face matching (the decision-making processes used to determine whether two or more face images are of the same individual, or different individuals), and face matching *tests*, which we have argued require the face matching psychological process but also face perception. It is our hypothesis that the psychological process of face matching is identical irrespective of whether one of the two faces is stored in memory or both faces are presented perceptually, and thus we suggest that the process of face matching is required for tests of both face recognition and face matching.

When face matching tasks are not used to assess face perception, the CFPT (Duchaine, Germine & Nakayama, 2007) is often used. The CFPT requires participants to arrange a set of morphed test face stimuli in order of their similarity to a target face. The extent to which face matching contributes to CFPT is difficult to determine given that test faces are themselves morphs of the target face. Thus, test faces that are most perceptually similar to the target face contain objectively more of the test face. This means that face matching, determining whether the target and test faces are the same identity, could contribute to successful task completion.

Ideally, one would obtain independent measures of face perception, face matching and face memory, and determine the degree to which individual differences in face perception and face matching explain variance in face memory. We suggest that this can be achieved through the use of the Oxford Face Matching Test (OFMT; Stantić, Brewer, et al., 2022a) alongside the CFMT, and have previously used this technique to derive independent measures of face perception, face matching and face memory, and to demonstrate that individuals with developmental prosopagnosia are impaired in all three face processes (Stantić, Pounder, et al., 2022b).

It is possible to derive independent measures of face processes because the OFMT is a face matching task that requires participants to make two responses. The first to judge the degree of perceptual similarity between two concurrently presented faces, and the second to decide whether the faces are the same or different. The OFMT is deliberately constructed such that faces in ‘match’ trials (where the faces are of the same identity) and ‘mismatch’ trials (faces are of different identities) contain overlapping similarity distributions – two images of the same person can be perceptually markedly different, while images of two different individuals can be perceptually very similar (e.g., Mileva et al., 2019; Sutherland et al., 2017; Todorov & Porter, 2014) – in order to aid dissociation of face perception and matching. Importantly, perceptual similarity of the two face images is established using three leading face recognition algorithms (see Stantić, Brewer, et al., 2022a). The three algorithms provide face similarity scores, with the mean similarity rating taken as the degree of face similarity. This mean algorithmic similarity value can be compared to the similarity rating provided by participants, to produce a face perception score. Algorithmic similarity is used because (1) such algorithms regularly outperform human observers (Phillips et al., 2018), and (2) use of algorithms to determine similarity rather than large groups of human raters avoids a systemic bias towards whichever groups rate the stimuli. With respect to the latter point, a number of groups of individuals are thought to exhibit atypical face processing, including individuals diagnosed with autism (Hedley et al., 2011; Wallace et al., 2008). If similarity between face pairs is decided on the basis of ratings by neurotypical, non-autistic individuals, then such ratings are systematically biased towards those neurotypical groups.

Although it is commonly recognised that (on average) face processing is impaired in autistic individuals,^1^ the particular sub-processes that may be impaired in autism are debated, with some authors arguing for a specific deficit in face memory (in the presence of intact face perception; Weigelt et al., 2012) and some arguing for impairments in both face perception and face memory (Griffin et al., 2021; Tang et al., 2015). To our knowledge, no previous study has assessed face matching independent of face perception in autism, nor face memory independent of face perception and matching. Accordingly, this paper reports two studies that aim to use the OFMT to determine the degree to which variance in face perception and face matching explain variance in face memory in neurotypical and autistic individuals, and to determine which aspects of face processing are impaired in autistic individuals – particularly whether face memory is impaired in autism after accounting for any impairment in face perception and face matching.

## Study 1

Study 1 attempted to determine the contributions of face perception and face matching to performance on the CFMT in a large sample of neurotypical individuals.

### Methods

#### Participants

The study included 1,189 adults, who took part in the experiment online; 77 were excluded based on predefined exclusion criteria (30 for failing attention checks, 23 for not finishing all tasks, four for data corruption, and 20 for scoring more than 3 standard deviations below the group mean on any task). Data are reported from the remaining sample of 1,112 participants (743 female, *M*_age_ = 37.60 years, *SD =* 18.98). Post hoc power analyses indicated that for the main analysis of interest (linear regression with two predictors) and a sample size of 1,112 participants, the smallest effect size for which there was 80% power at an alpha of .05 is an f^2^ of 0.0085. Participants were recruited from a variety of sources, including physical and social media advertisements, and via Prolific.co.

#### Procedure

Participants completed three previously validated measures of face processing ability in a randomized order: two matching paradigms, the OFMT (Stantić, Brewer, et al., 2022a) and the GFMT (Burton et al., 2010), and a face memory task, the CFMT (Duchaine & Nakayama, 2006). The study was approved by the local research ethics committee, and all authors report no conflicts of interest.

#### Oxford Face Matching Test (Stantić, Brewer, et al., 2022a)

**Fig. 1:**
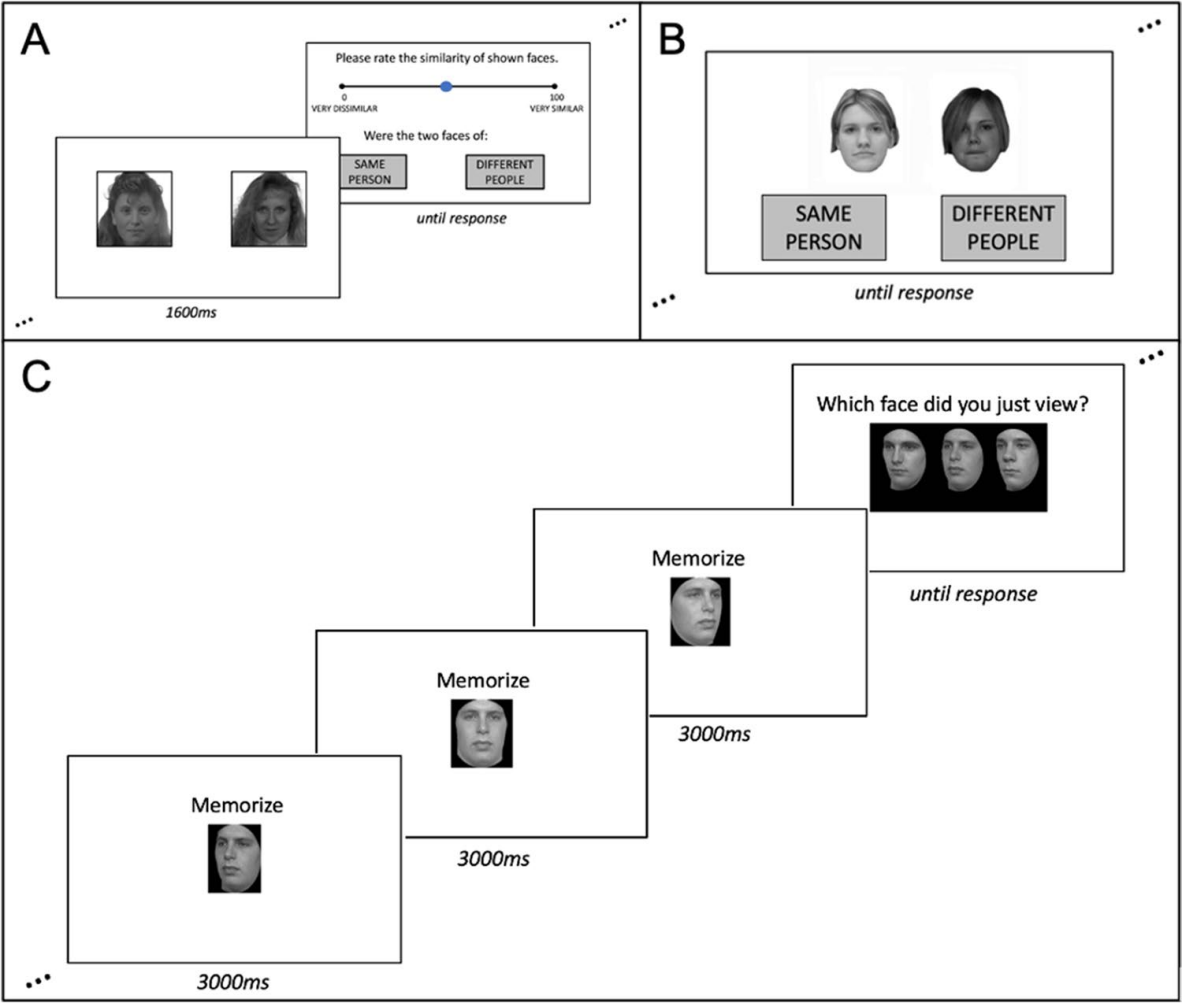
A sample trial of three face processing tasks: **A** – the Oxford Face Matching Test (OFMT), a face matching task that presents faces for 1,600 ms before participants have to rate the similarity of two faces and decide whether the faces are of the same person or of different people; **B** – the Glasgow Face Matching Test (GFMT), a face matching task that presents faces for an unlimited amount of time while participants decide whether the faces are of the same person or different people; and **C** – the Cambridge Face Memory Test (CFMT), a face memory task in which participants learn faces from three viewpoints and subsequently select them from test displays with two distractors

The OFMT (Fig. 1A) is a novel face matching task that contains 200 trials (100 match (same) and 100 mismatch (different) face pairs). Participants are presented with a face pair for 1,600 ms and asked to determine whether faces are of the same person or different people. Participants also provide a perceptual similarity judgment for each pair of faces on a scale from 0 to 100. The maximum possible matching accuracy score is 200.

#### Glasgow Face Matching Test (Burton et al., 2010)

The GFMT (Fig. 1B) is an established measure of face matching that contains 40 trials (20 match and 20 mismatch). Participants are presented with face pairs and are able to view them for an unlimited amount of time before making a decision about whether the faces are the same or different. The maximum possible score is 40.

#### Cambridge Face Matching Test (Duchaine & Nakayama, 2006)

The CFMT (Fig. 1C) is an established measure of face memory that contains 72 trials in three stages of increasing difficulty. Participants initially learn six faces and are afterwards tested on three-alternative forced-choice trials with two distractors and one image of a previously learnt identity. There are 18 trials with no changes to viewpoint or lighting, 30 trials with changes to viewpoint and lighting, and 24 trials with changes to viewpoint and lighting as well as the addition of visual noise. The maximum possible score is 72.

##### Analysis strategy

To derive a measure of face perception, participants’ ratings of the similarity of face pairs on the OFMT were compared to the algorithmically derived similarity ratings. Specifically, for each participant, an average absolute deviation from algorithmic similarity was calculated, such that the greater the deviation, the worse the face perception ability. The hypothesised contribution of face perception to face matching can be tested by regressing OFMT face matching accuracy scores on Deviation scores (derived from the OFMT). As a robustness check, GFMT matching accuracy scores can also be regressed onto the OFMT Deviation scores. Crucially, the independent contributions of face perception and face matching to face memory (in Study 1, ‘face memory’ refers to performance on the CFMT) can be established by regressing CFMT scores on Deviation scores and OFMT matching scores. This analysis allows the independent contribution of face matching to be established while controlling for (holding constant) face perception, and vice versa. Again, face matching scores from the OFMT can be replaced by those from the GFMT as a data robustness check. All data were analysed using SPSS Statistics Version 26.

### Results

**Fig. 2:**
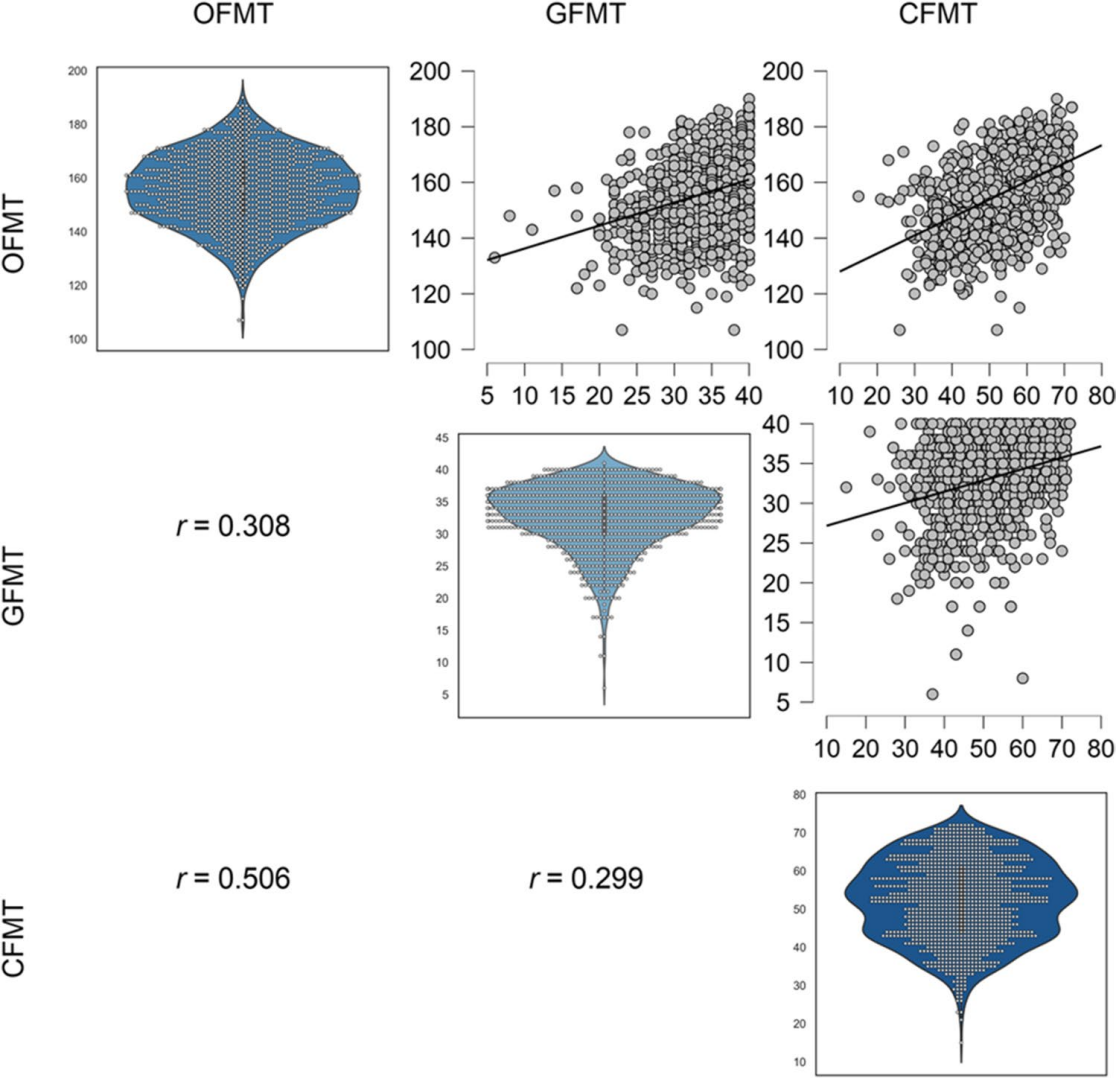
Correlations between all measures of face processing, along with the distributions of scores across all participants for all three tests. *OFMT* – the Oxford Face Matching Test (matching accuracy), *GFMT* – the Glasgow Face Matching Test, *CFMT* – the Cambridge Face Memory Test. All correlations are significant at p < .001

**Fig. 3:**
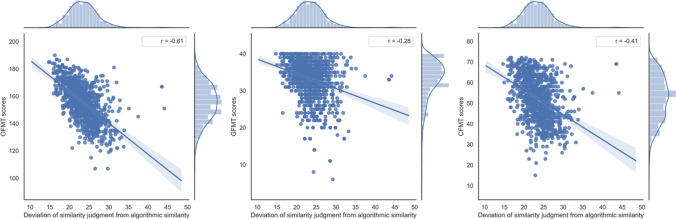
Linear regressions showing relationships between the deviation of individual participants’ similarity judgments across 200 trials of the OFMT from algorithmic similarity values, and the face matching and memory tasks. Left panel – relationship with the OFMT matching scores, middle panel – relationship with the GFMT scores, right panel – relationship with the CFMT scores. *OFMT* – the Oxford Face Matching Test (matching accuracy), *GFMT* – the Glasgow Face Matching Test, *CFMT* – the Cambridge Face Memory Test

Descriptive statistics for all measures are shown in Table 1, and descriptive statistics for Deviation scores are shown in Fig. 3. The distribution of scores across all participants is shown in Fig. 2 for all three tests, along with correlations between test scores.

**Table 1:** Descriptive statistics for all three face processing measures

	*n*	*M*	*SD*	Range
OFMT	1112	155.44	13.36	107-190
CFMT	1112	52.34	10.44	15-72
GFMT	1112	33.22	4.98	6-40

*OFMT* – Oxford Face Matching Test (matching accuracy), *CFMT* – Cambridge Face Memory Test, *GFMT* – Glasgow Face Matching Test

#### Contribution of face perception to face matching

Face perception ability, as measured by Deviation scores from algorithmic similarity judgements, was found to be a significant predictor of OFMT matching accuracy (β = -.611, t = 25.71, *p* <.001), such that individuals whose similarity estimates deviated more from the algorithms’ judgement were also worse at judging whether two images were of the same face, or different faces, on the OFMT. See Fig. 3A, left panel.

As a robustness check, the same analysis was performed on GFMT matching accuracy and yielded the same pattern of results. Deviation scores were found to be a significant predictor of GFMT scores (*β* = -.284, *t* = 9.88, *p* < .001), such that individuals whose estimates deviated more from the algorithms’ judgement on the OFMT were also worse at judging whether two images were of the same face, or different faces, on the GFMT. See Fig. 3A, middle panel.

#### Independent effects of face perception and face matching on face memory

CFMT scores were regressed onto Deviation scores (our measure of face perception) and face matching accuracy scores derived from the OFMT. A significant overall model accounted for 27.3% of the variance in CFMT scores, *F*(2, 1109) = 208.54, *p* < .001. Deviation scores were found to be a significant predictor of CFMT scores (*β* = -.165, *t* = 5.11, *p* < .001), such that individuals whose estimates deviated more from the algorithms’ judgement had worse face memory as measured by the CFMT (see Fig. 3A, right panel). OFMT face matching accuracy scores were also a significant independent predictor of CFMT scores (*β* = .405, *t* = 12.53, *p* < .001), such that those with a better ability to determine whether two faces are the same or different had better face memory as measured by the CFMT (see Fig. 3B, left panel). Again, as a robustness check, face matching accuracy scores derived from the OFMT were replaced with face matching accuracy scores derived from the GFMT and the same pattern of significance was observed (Deviations: *β* = -.357, *t* = 12.78, *p* < .001; Face Matching: *β* = .198, *t* = 7.08, *p* < .001).

### Discussion

In order to successfully recognise a previously learnt face, one must retrieve a stored representation of that face, assess the degree of similarity between the stimulus face and the stored face representation, and then decide whether the degree (and nature) of similarity is such that the stimulus and stored faces match, requiring accurate perceptual representations of the faces. Here, we attempted to test the independent contributions of face perception and face matching to face memory.

Face perception was assessed by comparing participants’ judgements of the similarity of two faces with a decision derived from three leading face recognition algorithms. This measure of face perception predicted face matching performance (i.e., the accuracy of the decision as to whether two images were of the same face or different faces), and it did so on two independent tests of face matching, the OFMT and GFMT. Face perception also predicted performance on the CFMT, a test of face memory. Face matching, assessed independently of face perception, also predicted face memory, irrespective of whether the OFMT or GFMT was used to derive the measure of face matching. These results are consistent with the hypothesised role of both face perception and face matching in face memory. Results are also consistent with the idea that the psychological process of face matching – deciding whether two faces are the same or different – is identical whether making a decision about two faces presented perceptually (as in the OFMT and GFMT) or when deciding whether a face presented perceptually matches that stored in memory (as in the CFMT). The fact that matching accuracy derived from both the OFMT and GFMT after accounting for face perception predicted CFMT, suggests the same process plays a role in all three tests.

Use of the OFMT and the analysis strategy adopted here allowed face matching ability to be assessed independent of face perception. In addition to face perception and face memory, this allows a further feature of individual differences in face processing to be measured. All three metrics can be used to assess the face processing abilities of different groups such as older adults (Stantić, Hearne, et al., 2021a), individuals with developmental prosopagnosia (Stantić, Pounder, et al., 2022b) or superior face recognition ('super recognisers’; Stantić et al., under review) and, as assessed in Study 2, individuals with autism.

## Study 2

As reported above, although it is commonly recognised that (on average) face processing is impaired in autistic individuals, the particular sub-processes which may be impaired in autism are debated, with some authors arguing for a specific deficit in face memory (in the presence of intact face perception; Weigelt et al., 2012) and some arguing for impairments in both face perception and face memory (Griffin et al., 2021; Tang et al., 2015). To our knowledge, no previous study has assessed face *matching* independent of face *perception* in autism, and it is possible that an impairment in face matching produces what appear to be deficits in face perception and memory in autism (Robel et al., 2004; Stantić, Ichijo, et al., 2021b).

### Methods

#### Participants

An opportunity sample was used whereby all participants registered on a database of autistic volunteers maintained by the authors were invited to take part, and a neurotypical sample recruited to match the autistic sample. Fifty-eight autistic individuals were recruited to take part, but one participant was excluded for failing to pass attention checks. The final sample consisted of 57 autistic individuals (32 male; *M*_age_ = 42.02 years, *SD =* 13.39), and 57 individuals with no current or previous clinical diagnosis of autism (29 male; *M*_age_ = 41.81 years, *SD* = 9.27). Neurotypical participants were recruited via Prolific.co. Post hoc power analyses indicated that for the main analysis of interest (linear regression with five predictors) and a sample size of 114 participants, the smallest effect size for which there was at least 50% power at an alpha of .05 is an R^2^ of 0.0606183. Autistic individuals were diagnosed by an independent clinician and all but two met criteria for autism or autism spectrum on the Autism Diagnostic Observation Schedule, Second Edition (ADOS-2; Lord et al., 2012). As expected, the autistic group had greater levels of autistic traits than the neurotypical group [*t*(105) = 9.84, *p* < .001], as assessed by the Autism-Spectrum Quotient (AQ-50; Baron-Cohen et al., 2001). However, the autistic and neurotypical groups did not differ significantly in terms of age [*t*(112) = 0.10, *p* = .922], gender [*X*^*2*^(1) = 0.32, *p* = .573], or non-verbal IQ [*t*(112) = 0.73, *p* = .468] as measured by the matrix reasoning component of the Wechsler Abbreviated Scale of Intelligence, Second Edition (WASI-II; Wechsler, 2011). Note that a subset of these data has previously been reported in Stantić, Ichijo, et al. (2021b).

#### Procedure

The procedure of Study 2 was as Study 1, with the addition of the matrix reasoning subtest of the WASI-II IQ test, and the AQ-50, a measure of autistic traits (Baron-Cohen et al., 2001).

#### Analysis strategy

Study 2 adopted the same analysis strategy as Study 1 for the main analysis, except that the variable Group (Autism vs. Neurotypical), and interactions with Group were included in regression models. Including the interaction term in regression models allows for the relationship between, for example, face perception and face matching, to vary across groups.

### Results

#### Group comparisons

The autistic group performed significantly worse than the neurotypical control group on all tests.

##### OFMT deviations

The Deviation scores of the autistic group (*M =* 26.37, *SD* = 4.12, range = 17.76–40.57) were significantly worse than the Deviation scores of the neurotypical group (*M =* 24.42, *SD* = 3.43, range = 16.14–30.96), *t*(112) = 2.75, *p* = .007. Thirty-nine out of 57 autistic participants (68.42%) scored above the median neurotypical score (indicating worse performance). Thus, the Autism group exhibited significantly worse face perception than the Neurotypical group.

##### OFMT matching accuracy

The performance of the autistic group *(M =* 138.42, *SD* = 12.41, range = 102–165) was significantly worse than the neurotypical group (*M =* 145.02, *SD* = 12.30, range = 112–168), *t*(112) = 2.85, *p* = .005. Forty of 57 autistic participants (70.02%) scored below the median neurotypical performance (indicating worse performance).

##### GFMT matching accuracy

A Shapiro-Wilk test of GFMT scores revealed a significant departure from normality, *W* = 0.96, *p* = .002. Therefore, a square transformation was applied to the total GFMT scores for statistical analysis. The performance of the autistic group *(M =* 30.75, *SD* = 5.23, range = 17–39, untransformed) was significantly worse than the neurotypical group (*M =* 33.16, *SD* = 3.84, range = 24–40, untransformed), *t*(112) = 2.69, *p* = .008. Thirty-five of 57 autistic participants (61.40%) scored below the median neurotypical performance (indicating worse performance).

##### CFMT

The performance of the autistic group *(M =* 46.54, *SD* = 10.88, range = 19–71) was significantly worse than the neurotypical group (*M =* 53.81, *SD* = 9.89, range = 36–72), *t*(112) = 3.73, *p* < .001. Forty-four of 57 autistic participants (77.19%) scored below the median neurotypical performance (indicating worse performance).

##### Face matching controlling for face perception

Group (Autism vs. Neurotypical), Deviation scores, and their interaction were entered into two regressions, one predicting OFMT matching accuracy and the other GFMT matching accuracy. For the OFMT analysis, Deviation scores were a significant predictor (again showing that face perception abilities are related to face matching performance; *β* = -.611, *t* = 8.00, *p* < .001), group was not a significant predictor (suggesting that face matching by autistic individuals was no different from neurotypical individuals after accounting for face perception; *β* = -.107, *t* = 1.42, *p* = .158), and the interaction between Group and Deviation scores was not a significant predictor (indicating that the relationship between face perception and OFMT matching accuracy did not vary as a function of group; *β* = -.009, *t* = 0.12, *p* = .908). For the GFMT analysis, the pattern of significance was the same. Deviation scores were a significant predictor (*β* = -.456, *t* = 5.41, *p* < .001), group was not a significant predictor (*β* = -.132, *t* = 1.59, *p* = .115), and the interaction between Group and Deviation scores was not a significant predictor (*β* = -.134, *t* = 1.64, *p* = .103).

##### Face memory controlling for face perception and face matching

Group (Autism vs. Neurotypical), Deviation scores, Face Matching (OFMT matching accuracy and separately GFMT matching accuracy) and the interactions between Deviation scores and Group, and Face Matching and Group, were entered into a regression predicting CFMT scores. For the OFMT analysis, results demonstrated significant independent contributions of face perception (as measured by Deviation scores), and face matching (*β* = -.248, *t* = 2.44, *p* = .016; *β* = .319, *t* = 3.14, *p* = .002, respectively). Group was a significant predictor (*β* = -.187, *t* = 2.33, *p* = .022), indicating that face memory was worse in autism even after accounting for face perception and face matching. The two interactions were not significant predictors, indicating the relationship between face memory and face perception and face matching did not vary as a function of group (Group × Deviation scores: *β* = -.044, *t* = 0.45, *p* = .657; Group × Face Matching: *β* = -.016, *t* = 0.167, *p* = .868).

For the GFMT analysis, the pattern of significance was identical. Results demonstrated significant independent contributions of face perception (as measured by Deviation scores), and face matching (*β* = -.291, *t* = 3.28, *p* = .001; *β* = .365, *t* = 4.12, *p* < .001, respectively). Group was a significant predictor (*β* = -.169, *t* = 2.17, *p* = .032), while the two interactions were not significant predictors (Group × Deviation scores: *β* = -.034, *t* = 0.391, *p* = .697; Group × Face Matching: *β* = -.101, *t* = 1.17, *p* = .245).

### Discussion

Study 2 revealed that the group of autistic individuals performed worse on all three tests of face processing (OFMT, GFMT and CFMT), and were worse at judging the similarity of two faces, than a matched control group. However, when face perception was controlled for by accounting for the degree to which similarity judgements deviated from algorithmic judgements, the group of autistic individuals did not perform worse than the control group on face matching whether measured using either the OFMT or the GFMT. This pattern of results indicated that although face perception is, on average, impaired in the autistic group, face matching is not. The poor performance on the CFMT (a test of face memory) has been shown previously (Griffin et al., 2021) and is normally taken as evidence of impaired face memory in autism. These results go further, in demonstrating an autistic impairment in face memory even after accounting for face perception and face matching. Overall then, results suggests that individuals with autism exhibit impaired face perception and face memory, but intact face matching.

These conclusions are consistent with the latest meta-analysis of face processing in autism (Griffin et al., 2021) in showing face perception and face memory difficulties in autism, but also show that the face memory impairment is not an artifact of face perception difficulties. In addition, and to our knowledge for the first time, these results show intact face matching ability in autism. In other words, given accurate perceptual representations, autistic individuals are no worse than neurotypical individuals in deciding whether two images are of the same face or different faces. Intact face matching in autism means that if face perception could be improved through clinical intervention, improved face recognition should follow (based on the results of Study 1). It should be noted, however, that a high degree of heterogeneity was observed in the autism group (Stantić, Ichijo, et al., 2021b). A proportion of autistic individuals (between one- and two-fifths of the sample) performed better than the median neurotypical individual, and some performed excellently. Such variation in performance is typically observed across the population of individuals with autism, across a range of cognitive tasks (Happé, Ronald & Plomin, 2006).

Interestingly, the general pattern of impairments in autism did not match those seen in developmental prosopagnosia (Stantić, Pounder, et al., 2022b), despite both autism and developmental prosopagnosia frequently being described as associated with face processing impairments. In autism, only face perception and face memory were impaired, but in developmental prosopagnosia all three processes (face perception, matching and memory) were impaired. This pattern highlights the clinical utility in adopting a cognitive framework to understand the difficulties faced by diverse groups of individuals. It is hoped that by adopting such an approach, alongside precise and accurate measurement of cognitive processes, more effective interventions may be derived (Frith, 2012).

Finally, it is worth considering whether one can really distinguish between face perception and face matching using the approach adopted in this study. It is clear that one can distinguish between face perception and face matching at the conceptual level, and it is also possible to conceive of individual differences in these processes arising from different sources. Individual differences in face *perception* could arise from difficulties that might be associated with building a three-dimensional face representation from degraded input from poor lighting, short exposures, low-resolution 2-D images, or in the presence of visual noise. Good face perceivers would be more likely to build accurate face models from degraded input than poor face perceivers. We suggest individual differences in face *matching* relate to (1) accurate models of how much, and in what dimensions, faces are allowed to vary before they must be judged to be different faces, and (2) appropriate use of these models when making decisions about facial identities. With these definitions one can imagine two equally good face perceivers who perform at markedly different levels on face matching tests because one does, and one does not, have an accurate model indicating that variation in skin colour, weight, and facial texture do not necessarily mean two faces are different, but that variation in the skeletal structure of the face likely does mean that two faces are different. Conversely, one can imagine two individuals who understand how faces vary within and between individuals equally well, and so are equally good at face matching, but one can, and the other cannot, construct accurate three-dimensional models of faces under non-optimal conditions.

These results, and those of Stantić, Brewer, et al. (2022a); Stantić, Pounder, et al. (2022b) using the same approach, show that face perception and face matching can be dissociated at the empirical level; groups can exhibit impairments in face perception without impairments in face matching (autism), and that these results are not an artefact of the analysis method, as other groups exhibit impairments in both processes (developmental prosopagnosia). However, measurement of face matching using this approach does rest on the assumption that there is nothing other than face matching and face perception that contributes to performance on face matching tasks, and this is unlikely to be true. One can imagine that working memory, vocabulary, or attentional control may all play a role in face matching performance and, although IQ is controlled in this study, future work should adopt an even more fine-grained approach to build ever more realistic models of individual differences in face processing.

## Data Availability

See Open Practices Statement.

## Footnotes

1 To respect the wishes of autistic individuals and report the study in line with scientific parlance, we use language preferred by clinical professionals (e.g., 'individuals with autism’), as well as the term ‘autistic’, a term endorsed by many individuals with ASD (see Kenny et al., 2016).
